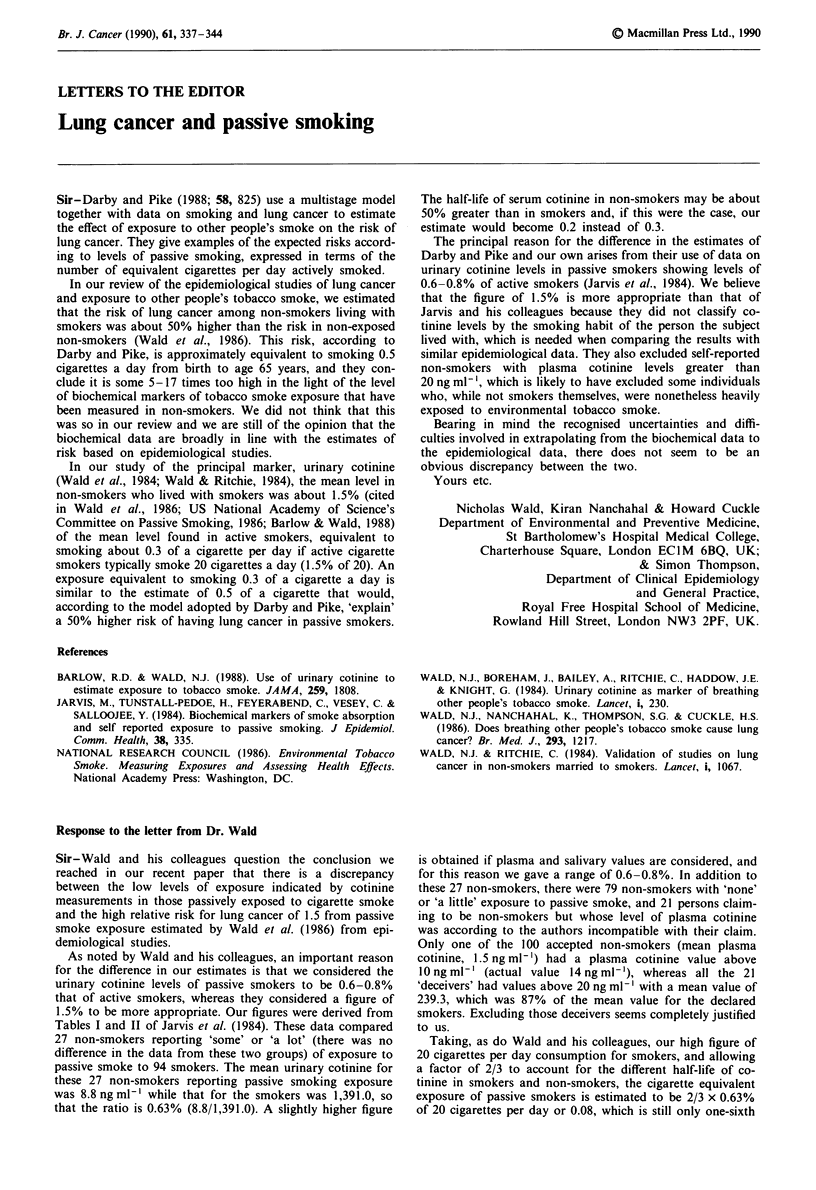# Lung cancer and passive smoking.

**DOI:** 10.1038/bjc.1990.67

**Published:** 1990-02

**Authors:** N. Wald, K. Nanchahal, H. Cuckle, S. Thompson


### LETTERS TO THE EDITOR

### Lung cancer and passive smoking

**Sir**–Darby and Pike (1988; **58**, 825) use a multistage model together with data on smoking and lung cancer to estimate the effect of exposure to other people's smoke on the risk of lung cancer. They give examples of the expected risks according to levels of passive smoking, expressed in terms of the number of equivalent cigarettes per day actively smoked.

In our review of the epidemiological studies of lung cancer and exposure to other people's tobacco smoke, we estimated that the risk of lung cancer among non-smokers living with smokers was about 50% higher than the risk in non-exposed non-smokers (Wald *et al.*, 1986). This risk, according to Darby and Pike, is approximately equivalent to smoking 0.5 cigarettes a day from birth to age 65 years, and they conclude it is some 5–17 times too high in the light of the level of biochemical markers of tobacco smoke exposure that have been measured in non-smokers. We did not think that this was so in our review and we are still of the opinion that the biochemical data are broadly in line with the estimates of risk based on epidemiological studies.

In our study of the principal marker, urinary cotinine (Wald *et al.*, 1984; Wald & Ritchie, 1984), the mean level in non-smokers who lived with smokers was about 1.5% (cited in Wald *et al.*, 1986; US National Academy of Science's Committee on Passive Smoking, 1986; Barlow & Wald, 1988) of the mean level found in active smokers, equivalent to smoking about 0.3 of a cigarette per day if active cigarette smokers typically smoke 20 cigarettes a day (1.5% of 20). An exposure equivalent to smoking 0.3 of a cigarette a day is similar to the estimate of 0.5 of a cigarette that would, according to the model adopted by Darby and Pike, 'explain' a 50% higher risk of having lung cancer in passive smokers. The half-life of serum cotinine in non-smokers may be about 50% greater than in smokers and, if this were the case, our estimate would become 0.2 instead of 0.3.

The principal reason for the difference in the estimates of Darby and Pike and our own arises from their use of data on urinary cotinine levels in passive smokers showing levels of 0.6–0.8% of active smokers (Jarvis *et al.*, 1984). We believe that the figure of 1.5% is more appropriate than that of Jarvis and his colleagues because they did not classify cotinine levels by the smoking habit of the person the subject lived with, which is needed when comparing the results with similar epidemiological data. They also excluded self-reported non-smokers with plasma cotinine levels greater than 20 ng $ml^{-1}$, which is likely to have excluded some individuals who, while not smokers themselves, were nonetheless heavily exposed to environmental tobacco smoke.

Bearing in mind the recognised uncertainties and difficulties involved in extrapolating from the biochemical data to the epidemiological data, there does not seem to be an obvious discrepancy between the two.

Yours etc.

Nicholas Wald, Kiran Nanchahal & Howard Cuckle
Department of Environmental and Preventive Medicine,
St Bartholomew's Hospital Medical College,
Charterhouse Square, London EC1M 6BQ, UK;
& Simon Thompson,
Department of Clinical Epidemiology
and General Practice,
Royal Free Hospital School of Medicine,
Rowland Hill Street, London NW3 2PF, UK.

### References

BARLOW, R.D. & WALD, N.J. (1988). Use of urinary cotinine to estimate exposure to tobacco smoke. *JAMA*, **259**, 1808.

JARVIS, M., TUNSTALL-PEDOE, H., FEYERABEND, C., VESEY, C. & SALLOOJEE, Y. (1984). Biochemical markers of smoke absorption and self reported exposure to passive smoking. *J Epidemiol. Comm. Health*, **38**, 335.

NATIONAL RESEARCH COUNCIL (1986). *Environmental Tobacco Smoke. Measuring Exposures and Assessing Health Effects.* National Academy Press: Washington, DC.

WALD, N.J., BOREHAM, J., BAILEY, A., RITCHIE, C., HADDOW, J.E. & KNIGHT, G. (1984). Urinary cotinine as marker of breathing other people's tobacco smoke. *Lancet*, **i**, 230.

WALD, N.J., NANCHAHAL, K., THOMPSON, S.G. & CUCKLE, H.S. (1986). Does breathing other people's tobacco smoke cause lung cancer? *Br. Med. J.*, **293**, 1217.

WALD, N.J. & RITCHIE, C. (1984). Validation of studies on lung cancer in non-smokers married to smokers. *Lancet*, **i**, 1067.